# *FUT2* secretor genotype and susceptibility to infections and chronic conditions in the ALSPAC cohort

**DOI:** 10.12688/wellcomeopenres.14636.2

**Published:** 2018-09-25

**Authors:** Meghan B. Azad, Kaitlin H. Wade, Nicholas J. Timpson

**Affiliations:** 1 Manitoba Developmental Origins of Chronic Diseases in Children Network (DEVOTION), Children’s Hospital Research Institute of Manitoba, Department of Pediatrics and Child Health, University of Manitoba, Winnipeg, R3E 3P4, Canada; 2 Medical Research Council Integrative Epidemiology Unit, Avon Longitudinal Study of Parents and Children, Population Health Science, Bristol Medical School, University of Bristol, Bristol, BS8 2BN, UK

**Keywords:** FUT2, infection, chronic disease, secretor status, ALSPAC, mumps, kidney disease

## Abstract

**Background: **The *FUT2 *(fucosyltransferase-2) gene determines blood group secretor status. Being homozygous for the inactive “non-secretor” rs601338(A) allele confers resistance to certain infections (e.g. *Norovirus*, *Rotavirus*) and susceptibility to others (e.g. *Haemophilus influenza*, *Streptococcus pneumonia*). Non-secretors also have an increased risk of type 1 diabetes and inflammatory bowel disease. We examined *FUT2 *genotype, infections and chronic conditions in a population-based cohort.

**Methods: **We studied 7,582 pregnant women from the ALSPAC pregnancy cohort. Infections (measles, mumps, chicken pox, whooping cough, meningitis, herpes, gonorrhea and urinary infections) and chronic conditions (kidney disease, hypertension, diabetes, rheumatism, arthritis, psoriasis, hay fever, asthma, eczema and allergies) were self-reported. *FUT2* secretor status was determined from the rs601338 genotype. ABO blood type was obtained from clinical records.

**Results: **Overall, 1920 women (25.3%) were homozygous for the non-secretor allele (AA). Secretor status was associated with mumps, with 68% of non-secretors experiencing this infection, compared to 48% of secretors (RR, 1.40; 95% CI, 1.34–1.46). A weaker association was observed for measles infection (76% vs. 72%; RR, 1.05; 95% CI, 1.02–1.09). Non-secretors also experienced an increased risk of kidney disease (5.4% vs. 3.9%; RR, 1.39; 95% CI, 1.11–1.75). Independent of secretor status, AB blood type was a risk factor for mumps (RR 1.15; 95%CI, 1.03, 1.28 compared to type O). We found no evidence of interaction between secretor status and blood type.  For some conditions, including asthma and arthritis, *FUT2 *heterozygosity (GA) appeared to confer an intermediate phenotype. There was no strong evidence of association between secretor status and other infections or chronic conditions, although statistical power was limited for rare outcomes.

**Conclusion: **Our results identify an association between *FUT2 *secretor status and self-reported kidney disease, and confirm a recently reported association with susceptibility to mumps infection. The clinical implications of these associations warrant further investigation.

## Introduction

The *FUT2* (fucosyltransferase 2) gene encodes the alpha (1,2) fucosyltransferase, which determines blood group secretor status. About 20% of Caucasians are homozygous for the nonsense mutation W143X (rs601338G>A), encoding a stop codon that inactivates the *FUT2* enzyme ^1^. Individuals who are homozygous for this “non-secretor” allele (AA) are unable to secrete histo-blood group antigens into bodily fluids, or express them on mucosal surfaces.

Non-secretors have a lower risk of diarrheal illness ^2^ and ear infections in childhood ^3^. The non-secretor phenotype also confers resistance to specific pathogens that require *FUT2*-dependent antigens to infect host cells, including *Norovirus* ^4– 7^, *Rotavirus* ^8– 11^ and *Helicobacter pylori* ^12, 13^. By contrast, the non-secretor phenotype has been associated with increased susceptibility to other pathogens, including *Candida* ^14– 16^, *Haemophilius influenza* ^17^, *Neisseria meningitis* ^18^ and *Streptococcus pneumonia* ^18^. Most recently, in a genome-wide association study (GWAS) of common infections, Tian *et al*. reported an increased susceptibility to mumps in non-secretors ^3^. In addition, non-secretors appear to be at increased risk for certain autoimmune diseases, including type 1 diabetes ^19^, psoriasis ^20, 21^ and inflammatory bowel disease ^22, 23^.

The above associations have not been simultaneously examined in a single population and several have not been independently replicated. Moreover, the association of *FUT2* secretor status with other infectious and chronic diseases has not been widely studied. Finally, previous studies have typically only considered the secretor phenotype as dichotomous, assuming the non-secretor allele to be recessive. In this study, we characterized the association of *FUT2* secretor status with a variety of infectious and chronic diseases in the population-based Avon Longitudinal Study of Parents and Children (ALSPAC), and examined the impact of heterozygosity for the non-secretor allele.

## Methods

### Study design and population

This study accessed data from the ALSPAC cohort. ALSPAC recruited 14,541 pregnant women (98% Caucasian) resident in the former county of Avon, UK with expected dates of delivery 1st April 1991 to 31st December 1992 ^24, 25^. The current analysis included a subset of 7,582 Caucasian women who selected and provided written informed consent for genotyping analysis, and reported their personal medical history during pregnancy. ABO blood group was collected from clinical records for the majority of participants (N=6,757). The ALSPAC website contains details of all the data that is available through a fully searchable data dictionary at http://www.bris.ac.uk/alspac/researchers/data-access/data-dictionary/. Ethical approval for the study was obtained from the ALSPAC Ethics and Law Committee and the Local Research Ethics Committees.

### Genotyping

ALSPAC mothers were genotyped using the Illumina human660W-quad array at Centre National de Génotypage (CNG) (Evry, France) and genotypes were called with Illumina GenomeStudio. PLINK (v1.07) was used to carry out quality control (QC) measures on an initial set of 10,015 participants and 557,124 directly genotyped single nucleotide polymorphisms (SNPs). SNPs were removed if they displayed more than 5% missingness or a Hardy-Weinberg equilibrium P value of less than 1.0×10 ^−6^. Additionally, SNPs with a minor allele frequency of less than 1% were removed. Samples were excluded if they displayed more than 5% missingness, had indeterminate X chromosome heterozygosity or extreme autosomal heterozygosity. Samples showing evidence of population stratification were identified by multidimensional scaling of genome-wide identity by state pairwise distances using the four HapMap populations as a reference, and then excluded ^26^. Cryptic relatedness was assessed using an identity by descent (IBD) estimate of more than 0.125, which is expected to correspond to roughly 12.5% alleles shared IBD or relatedness at the first cousin level. Related participants that passed all other QC thresholds were retained during subsequent phasing and imputation. In total, 9,048 mothers and 526,688 SNPs passed these QC filters.

### Imputation

A total of 477,482 SNP genotypes in common between the sample of mothers described above and a second sample of 9,115 children were combined. SNPs with genotype missingness above 1% due to poor quality (N=11,396 SNPs) were removed and a further 321 participants were removed due to potential ID mismatches. This resulted in a dataset of 17,842 participants, containing 6,305 duos and 465,740 SNPs (112 were removed during liftover and 234 were out of HWE after combination). Haplotypes were estimated using ShapeIT (v2.r644), which utilizes relatedness during phasing. The phased haplotypes were then imputed to the Haplotype Reference Consortium (HRC) panel of approximately 31,000 phased whole genomes using Impute V3. For this study, we excluded the mothers who had removed consent, leaving 8,698 eligible mothers. We further excluded those who did not provide personal medical history, leaving 7,582 for analysis.

### Exposure: FUT2 genotype

*FUT2* secretor status was defined based on the rs601338 SNP ^1^, where G is the wild-type “secretor” allele and A is the nonsense W143X “non-secretor” allele. Following previous studies ^7, 11, 19, 23^, we considered the A allele to be recessive and dichotomized secretor status, combining the GA and GG genotypes as secretors and comparing them to the homozygous AA non-secretors. In addition, we explored the impact of GA heterozygosity at this locus. Two other commonly reported non-secretor alleles were considered. The missense variant at rs1047781, described in Asian populations ^27^ was not detected in our Caucasian population. The non-synonymous S258G variant at rs602662 ^28^ was highly correlated with rs601338; incorporating this SNP to define secretor status had no impact on 98% of participants’ phenotype classification, and did not materially change our results.

### Outcomes: infections and chronic conditions

Infections and chronic conditions were self-reported using a standardized questionnaire during pregnancy. Women were asked if they had ever had various infections (measles, mumps, chicken pox, whooping cough, cold sores, meningitis, genital herpes, gonorrhea and urinary infections) or chronic conditions (diabetes, hypertension, kidney disease, rheumatism, arthritis, psoriasis, hay fever, asthma, eczema, and any allergies, including cat, dust, pollen, insect bites or ‘other’).

### Statistical analysis

Demographic characteristics were summarized with descriptive statistics and compared between non-secretors (AA) and secretors (GA or GG combined) using t-tests for continuous variables or chi-squared tests for categorical variables. For each outcome, the relative risk (RR) and 95% confidence interval (95% CI) was calculated for non-secretors versus secretors. A multivariable model was used to determine whether the association of *FUT2* secretor status and kidney disease was independent of measles, mumps and urinary tract infections. Multivariable models were also used to mutually adjust for *FUT2* secretor status and ABO blood group and to formally test for interaction between these two factors. To explore the potential impact of *FUT2* heterozygosity, a three group analysis was also conducted, considering the AA, GA and GG genotypes separately and using homozygous secretors (GG) as the reference group. All statistical analyses were performed in SAS (version 9.4, Carey, NC, US).

## Results

Overall, 1920 women were homozygous for the *FUT2* non-secretor allele (AA, 25.3%), 1906 were homozygous for the secretor allele (GG, 25.1%) and 3756 were heterozygous (GA, 49.5%). Almost half (46%) were first-time mothers, 21% were unmarried, 21% smoked, and 14% had a university degree. The mean (± standard deviation) age was 26.9 (± 5.9) years and the mean body mass index was 22.9 (± 3.7) kg/m ^2^. These demographic characteristics were not associated with *FUT2* secretor status ( Table 1). The lifetime incidence of infections ranged from <1% for meningitis to 87% for chicken pox, while the incidence of chronic conditions ranged from 1% for diabetes to 43% for allergies.

**Table 1.** Demographics of mothers in the ASLPAC cohort according to *FUT2* secretor status.

	*FUT2* Secretor Status (rs601338 genotype)				
	Non-Secretors (AA)		Secretors (GG or GA)		P value
	N=1920		N=5662		
Age, years	26.9	± 5.9	26.9	± 5.8	0.94
BMI, kg/m ^2^	22.8	± 3.6	23.0	± 3.7	0.05
Married					
No	423	(22.4)	1173	(21.2)	0.28
Yes	1468	(77.6)	4364	(78.8)	
Parity					
0	861	(46.3)	2539	(46.2)	0.67
1	641	(34.4)	1957	(35.6)	
2	264	(14.2)	738	(13.4)	
3 or more	95	(5.1)	259	(4.7)	
Smoking					
No	1480	(78.8)	4324	(77.9)	0.37
Yes	397	(21.2)	1229	(22.1)	
Education					
<O level	457	(24.6)	1418	(25.8)	0.44
O level	642	(34.5)	1946	(35.4)	
A level	461	(24.8)	1306	(23.8)	
University degree	276	(14.8)	762	(13.9)	

Values are mean ± standard deviation or n (%). AA, homozygous for non-secretor alleles; GG, homozygous for secretor alleles; GA, heterozygous; ALSPAC, Avon Longitudinal Study of Parents and Children; BMI, body mass index. Comparisons by t-test for continuous variables or chi-squared test for categorical variables.

### Dichotomous FUT2 secretor status and infections

The homozygous AA non-secretor genotype was associated with mumps infection, with 68% of non-secretors experiencing this infection, compared to 48% of secretors (RR, 1.40; 95% CI, 1.34–1.46; p<0.0001) ( Table 2). Weaker associations were observed for measles infection (76% vs. 72%; RR, 1.05; 95% CI, 1.02–1.09; p=0.0008) and urinary infections (57% vs. 55%; RR, 1.05; 95% CI, 1.00–1.10; p=0.05). There was no strong evidence of association between *FUT2* secretor status and whooping cough, chicken pox or cold sores ( Table 2).

**Table 2.** Lifetime incidence and relative risk of infectious and chronic conditions among mothers in the ALSPAC cohort according to dichotomized *FUT2* secretor status.

Condition *	*FUT2* Secretor Status (rs601338 genotype)				Relative Risk Non-Secretors vs. Secretors	
	Non-Secretors (AA)		Secretors (GA or GG)			P value
	N=1920		N=5662			
	cases	(%)	cases	(%)	RR (95% CI)	
Infections						
measles	1458	(75.9)	4076	(72.0)	**1.05 (1.02, 1.09)**	** 0.0008**
mumps	1299	(67.7)	2734	(48.3)	**1.40 (1.34, 1.46)**	**<0.0001**
chicken pox	1656	(86.3)	4925	(87.0)	0.99 (0.97, 1.01)	0.41
whooping cough	222	(11.6)	638	(11.3)	1.03 (0.89, 1.18)	0.73
cold sores	843	(43.9)	2458	(43.4)	1.01 (0.95, 1.07)	0.71
meningitis	15	(0.8)	61	(1.1)	0.73 (0.41, 1.27)	0.26
genital herpes	45	(2.3)	108	(1.9)	1.23 (0.87, 1.73)	0.24
gonorrhea	29	(1.5)	70	(1.2)	1.22 (0.80, 1.88)	0.36
urinary infection	1095	(57.0)	3085	(54.5)	**1.05 (1.00, 1.10)**	**0.05**
Chronic conditions						
kidney disease	103	(5.4)	218	(3.9)	**1.39 (1.11, 1.75)**	**0.004**
hypertension	272	(14.2)	804	(14.2)	1.00 (0.88, 1.13)	0.94
diabetes	23	(1.2)	55	(1.0)	1.23 (0.76, 2.00)	0.40
rheumatism	93	(4.8)	230	(4.1)	1.19 (0.94, 1.51)	0.14
arthritis	77	(4.0)	188	(3.3)	1.21 (0.93, 1.57)	0.15
psoriasis	59	(3.1)	213	(3.8)	0.82 (0.62, 1.08)	0.16
hay fever	573	(29.8)	1742	(30.8)	0.97 (0.90, 1.05)	0.45
asthma	215	(11.2)	652	(11.5)	0.97 (0.84, 1.12)	0.71
eczema	469	(24.4)	1271	(22.4)	1.09 (0.99, 1.19)	0.07
any allergies	837	(43.6)	2412	(42.6)	1.02 (0.97, 1.09)	0.42

AA, homozygous for non-secretor alleles; GG, homozygous for secretor alleles; GA, heterozygous; ALSPAC, Avon Longitudinal Study of Parents and Children; RR, relative risk; CI, confidence interval. *Self-reported during pregnancy: "Have you ever had…?"

### Dichotomous FUT2 secretor status and chronic conditions

Homozygous AA non-secretors experienced a 39% increased risk of self-reported kidney disease compared to secretors (5.4% vs. 3.9%; RR, 1.39; 95% CI, 1.11–1.75; p=0.004) ( Table 2). This association was essentially unchanged in a multivariable model controlling for mumps, measles and urinary infections (adjusted RR, 1.39; 95% CI, 1.10–1.75; p=0.005). Directionally consistent results were also observed for diabetes (RR, 1.23; 95% CI, 0.76–2.00; p=0.40), rheumatism (RR 1.19, 95%CI: 0.94–1.51, p=0.14) and arthritis (RR, 1.21; 95% CI, 0.93–1.57; p=0.15), although power was lacking for these relatively rare outcomes. There was no strong evidence of association between *FUT2* secretor status and hypertension, hay fever, asthma or allergies ( Table 2).

### ABO blood group

Since secretor status determines the ability to secrete blood group antigens, we also explored the impact of ABO blood group on associations observed for mumps infection and kidney disease ( Table 3). For both conditions, the effect estimate for *FUT2* secretor status was essentially unchanged following adjustment for ABO blood group and there was no significant interaction between *FUT2* and ABO blood group (p for interaction: 0.60 for mumps, 0.57 for kidney disease). Independent of *FUT2* secretor status, women with type AB blood had an increased risk of mumps infection (59.4%) compared to women with type A, B, or O blood (52.2%, 52.0%, 52.9%, respectively; adjusted RR 1.15, 95% CI: 1.03, 1.28 for AB vs O, p=0.01). ABO blood group was not associated with kidney disease.

**Table 3.** Mutually-adjusted associations of *FUT2* secretor status and ABO blood group with mumps infection and kidney disease in the ALSPAC cohort

	Mumps				Kidney Disease			
	n/N	%	RR (95% CI)	p	n/N	%	RR (95% CI)	p
***FUT2* Genotype**								
Non-Secretor (AA)	1299/1920	67.7	**1.39 (1.33, 1.46)**	**<.0001**	103/1920	5.4	**1.32 (1.04, 1.69)**	**0.02**
Secretor (AG or GG)	2734/5662	48.3	1.00 (ref)		218/5662	3.9	1.00 (ref)	
**Blood Group**								
A	1522/2917	52.2	1.00 (0.95, 1.05)	0.93	135/2917	4.6	1.09 (0.56, 2.11)	0.14
B	316/608	52.0	0.99 (0.91, 1.07)	0.78	26/608	4.3	1.10 (0.73, 1.67)	0.64
O	1595/3015	52.9	1.00 (ref)		117/3015	3.9	1.00 (ref)	
AB	129/217	59.4	**1.15 (1.03, 1.28)**	**0.01**	9/217	4.1	1.09 (0.56, 2.11)	0.80

RR, relative risk; CI, confidence interval. Models are mutually adjusted for *FUT2* genotype and blood group.

### FUT2 heterozygosity

**Figure 1.**
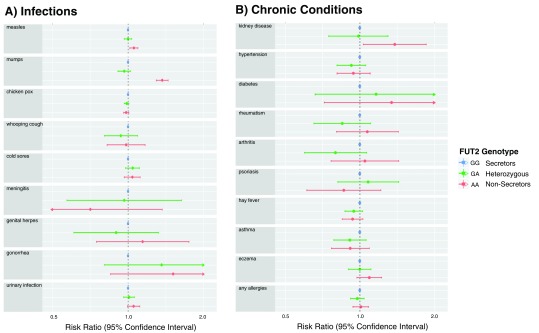
Relative risk of ( **A**) infectious and ( **B**) chronic conditions among 7,582 mothers in the ALSPAC cohort according to *FUT2* genotype at rs601338.

Compared to homozygous GG secretors, GA heterozygotes experienced a similar risk of mumps infection (RR, 0.97; 95% CI, 0.91–1.02; p=0.24) and kidney disease (RR, 0.99; 95% CI, 0.75–1.30; p=0.93), suggesting that increased susceptibility to these conditions (as described above) is likely to be a recessive trait experienced only in homozygous AA non-secretors. Similar evidence was found for measles, urinary infections and eczema, where disease risk was comparable for individuals with GG and GA genotypes. However, this pattern was not consistent across all conditions. For example, the risk of asthma was similarly reduced in GA heterozygotes (11.1%) and AA non-secretors (11.2%) compared to GG secretors (12.2%), and the risk of arthritis was lower in GA heterozygotes (3.0%) compared to either homozygous genotype (4.0% in AA, 3.8% in GG), although statistical evidence of association was weak for these relatively rare outcomes ( Figure 1).

## Discussion

Our findings from the population-based ALSPAC cohort confirm and extend previous research associating the *FUT2* genotype with susceptibility to infections and chronic diseases. Specifically, we confirmed a recently reported association with mumps infection ^3^ and identified an association with self-reported kidney disease. We also evaluated a number of other common conditions (e.g. whooping cough, chicken pox and asthma) but found no strong evidence of association with *FUT2* secretor status, indicating that *FUT2* influences pathogen- or disease-specific processes, rather than overall innate or adaptive immunity. Finally, our results suggest that *FUT2* heterozygosity may confer an intermediate phenotype for certain conditions, although further research is required to replicate these findings.

Our results confirm the association reported in a recent GWAS for common infections among 23andMe research participants by Tian *et al*. ^3^, where the *FUT2* rs516316(C) allele was associated with mumps infection (odds ratio, 1.25; 95% CI, 1.24–1.27). This risk allele is in complete linkage disequilibrium with the non-secretor rs601338(A) allele evaluated in our study, where a strong association was also observed (RR, 1.40; 95% CI, 1.34–1.46). Tian *et al*. hypothesized that non-secretors are more susceptible to mumps infection because binding of the mumps virus to host cell sialic acid receptors is enhanced in the absence of *FUT2-*dependent antigens on the cell surface. Indeed, using x-ray crystallography and functional assays, Kubota *et al.* ^29^ recently showed that mumps virus preferentially uses a trisaccharide containing α2,3-linked sialic acid in unbranched sugar chains as a receptor. Our results provide further evidence that susceptibility to mumps infection is modulated by *FUT2* secretor status.

Tian *et al*. ^3^ also reported an association between the ABO gene and mumps infection, and suggested that ABO antigens may disrupt binding of the mumps virus to host cell receptors. Our results using clinical ABO blood group data support and extend this finding by confirming an association and specifically identifying blood type AB as a risk factor for mumps infection. Since *FUT2* secretor status determines whether ABO antigens are secreted into body fluids and onto cell surfaces, we hypothesized that secretor status and blood type may interact to influence susceptibility to mumps infection; however, we found no evidence of this interaction. Thus, our study suggests that *FUT2* genotype and ABO blood group are independently associated with mumps infection, with increased risk among non-secretors and blood type AB.

Our study also provides new evidence that non-secretors may be predisposed to kidney disease (RR, 1.39; 95% CI, 1.11–1.75), although we lacked clinical information to confirm and classify this self-reported diagnosis. To our knowledge, the *FUT2* genotype has not previously been associated with kidney disease in the general population, although some studies have used traditional blood group assays to evaluate secretor status in patients with pyelonephritis (kidney inflammation, typically due to bacterial infection). One study of women with acute uncomplicated pyelonephritis found that non-secretor status was significantly more common in these patients than in the general population ^30^, and another found that renal scarring in girls with recurrent pyelonephritis was more common in non-secretors than secretors ^31^. It has been hypothesized that these associations reflect an increased susceptibility to uropathogenic *Escherichia coli* infection among non-secretors, resulting from the enhanced expression of preferred binding receptors in the vaginal epithelium and kidneys of non-secretor women ^32, 33^. Notably, in our study, the association between *FUT2* genotype and self-reported kidney disease appeared to be independent of self-reported urinary infections. However, there are multiple clinically-distinct causes of “kidney disease” and “urinary infections”, and we lacked clinical information to define the etiology of these conditions in our study. Thus, additional research is needed to replicate our observations with confirmed and clinically-defined kidney disease and urinary infections, and to examine the possible relationship between these conditions and *FUT2* genotype.

Consistent with previous studies ^19– 21^, we observed a trend towards an increased risk of arthritis, rheumatism and diabetes among non-secretors, although we lacked statistical power for the analysis of these relatively uncommon autoimmune disorders.

Finally, we examined disease risk among GA heterozygotes, who are typically considered secretors because the non-secretor rs601338(A) allele is assumed to be recessive. Our results for mumps and kidney disease support this assumption, as increased susceptibility was only seen in homozygous AA non-secretors. However, we observed different patterns of association for some other conditions, including a potentially increased risk of gonorrhea and reduced risk of arthritis among GA heterozygotes, although our effect estimates were imprecise for these relatively rare conditions. Further research is warranted to replicate these observations in larger populations, and explore whether heterozygosity may impart an intermediate risk or unique protection from certain conditions.

Limitations of this work include the reliance on self-reported medical histories and low power for rare outcomes (such as meningitis, diabetes and other autoimmune diseases). Power was also limited for interaction analyses. Also, we could not identify the specific pathogens responsible for urinary infections, and we lacked clinical data to confirm, classify and define the etiology of multifactorial disorders (such as allergies and kidney disease). Finally, our analysis of the ALSPAC pregnancy cohort was limited to women, so the results may not be generalizable to men, and potential sex differences could not be investigated.

In conclusion, our results identify a novel association between *FUT2* non-secretor status and increased risk of kidney disease, and confirm a recently-reported association with increased susceptibility to mumps infection. The clinical implications of these associations warrant further investigation.

## Data availability

The ALSPAC data management plan ( http://www.bristol.ac.uk/alspac/researchers/data-access/documents/alspac-data-management-plan.pdf) describes in detail the policy regarding data sharing, which is through a system of managed open access. The steps below highlight how to apply for access to the data included in this paper and all other ALSPAC data. The datasets used in this analysis are linked to ALSPAC project number B3047; please quote this project number during your application.

1. Please read the ALSPAC access policy (PDF, 627kB) which describes the process of accessing the data and samples in detail, and outlines the costs associated with doing so.
2. You may also find it useful to browse the fully searchable ALSPAC research proposals database, which lists all research projects that have been approved since April 2011.
3. Please submit your research proposal for consideration by the ALSPAC Executive Committee. You will receive a response within 10 working days to advise you whether your proposal has been approved.

If you have any questions about accessing data, please email alspac-data@bristol.ac.uk.
