# Supplementary material for: FUT2 secretor genotype and susceptibility to infections and chronic conditions in the ALSPAC cohort
Source: Wellcome Open Res. 2018 Sep 25;3:65. Originally published 2018 May 30. [Version 2] doi: 10.12688/wellcomeopenres.14636.2 (PMC6171556; doi:10.12688/wellcomeopenres.14636.2)
Supplement: Peer review history

# Peer review: Referee response for version 2

**DOI:** 10.21956/wellcomeopenres.16080.r33977

**Published:** 2018-09-27

**Authors:** Jacques Le Pendu

**Affiliations:** 1 CRCINA (Center for Research in Cancerology and Immunology Nantes-Angers), Inserm (French National Institute of Health and Medical Research), University of Angers, University of Nantes, Nantes, France

The authors have now adequately answered all of my queries.

I have read this submission. I believe that I have an appropriate level of expertise to confirm that it is of an acceptable scientific standard.

# Response

**Published:** 2018-09-28

**Authors:** Meghan Azad

**Affiliations:** University of Manitoba, Canada

Thank you Dr. Le Pendu for the helpful comments. We appreciate your input, and belive it has strengthened the paper.

# Peer review: Referee response for version 2

**DOI:** 10.21956/wellcomeopenres.16080.r33978

**Published:** 2018-09-26

**Authors:** Leonor David

**Affiliations:** 1 Differentiation and Cancer Group, Institute of Molecular Pathology and Immunology of the University of Porto/Institute for Research and Innovation in Health of University of Porto (IPATIMUP/i3S), Porto, Portugal

The authors addressed my comments thoroughly and I hope that contributed to improve the paper.

I have read this submission. I believe that I have an appropriate level of expertise to confirm that it is of an acceptable scientific standard.

# Response

**Published:** 2018-09-26

**Authors:** Meghan Azad

**Affiliations:** University of Manitoba, Canada

Thank you, Dr. David. The paper is indeed improved thanks to your comments. We appreciate your input!

# Peer review: Referee response for version 1

**DOI:** 10.21956/wellcomeopenres.15935.r33319

**Published:** 2018-06-29

**Authors:** Leonor David, Susana Seixas

**Affiliations:** 1 Differentiation and Cancer Group, Institute of Molecular Pathology and Immunology of the University of Porto/Institute for Research and Innovation in Health of University of Porto (IPATIMUP/i3S), Porto, Portugal; 2 IPATIMUP/I3S, Porto, Portugal

The paper entitled “FUT2 secretor genotype and susceptibility to infections and chronic conditions in the ALSPAC cohort”, identifies, in a cohort of 7,582 pregnant women, that the 25.3% homozygous for the FUT2 non-secretor allele have an increased incidence of mumps and a 39% increase in kidney disease. The association with mumps confirms a previous study by Tian et al, consolidates the implications of secretor status in this disease and increases the number of human infections linked to secretor status. We agree with Jacques Le Pendu on the interest to add the ABO status to enlarge the scope of the manuscript.

 The association with kidney disease is a weaker point of the paper. The diseases were based on self-reporting and can therefore represent a wide range of entities with different etiopathogenesis. The association was however unchanged when controlling for urinary infections, again self-reported, despite that there was a weak association with urinary infection. If there is no possibility to access clinical data to clarify the etiopathogenesis of the kidney disease then a more careful approach should be used in writing these results.

 The associations between FUT2 heterozygosity and gonorrhea, where heterozygotes behaved similarly to non-secretors, is not significant and the relative risk in Figure 1 includes large confidence intervals (from <1 to 2).  Also, the authors should not conclude that there is absence of recessivity since they did not exclude the presence of other null/nonfunctional FUT2 alleles. This hypothesis could be tested by excluding alternative mutations, namely rs1047781 and rs602662.

 In the analysis of population stratification the authors should explain why they used HapMap populations instead of 1000 Genomes, which includes more samples of the different human groups (European, Africans and Asians) and also have a deeper variation coverage.

We have read this submission. We believe that we have an appropriate level of expertise to confirm that it is of an acceptable scientific standard, however we have significant reservations, as outlined above.

# Response

**Published:** 2018-08-14

**Authors:** Meghan Azad

**Affiliations:** University of Manitoba, Canada

**Thank you for your comments, Dr. David. Please see our point-by-point reponses below in bold.**

The paper entitled “FUT2 secretor genotype and susceptibility to infections and chronic conditions in the ALSPAC cohort”, identifies, in a cohort of 7,582 pregnant women, that the 25.3% homozygous for the FUT2 non-secretor allele have an increased incidence of mumps and a 39% increase in kidney disease. The association with mumps confirms a previous study by Tian et al, consolidates the implications of secretor status in this disease and increases the number of human infections linked to secretor status. We agree with Jacques Le Pendu on the interest to add the ABO status to enlarge the scope of the manuscript.

**Please see our response to Dr. Le Pendu regarding ABO blood type. This has now been incorporated.**

The association with kidney disease is a weaker point of the paper. The diseases were based on self-reporting and can therefore represent a wide range of entities with different etiopathogenesis. The association was however unchanged when controlling for urinary infections, again self-reported, despite that there was a weak association with urinary infection. If there is no possibility to access clinical data to clarify the etiopathogenesis of the kidney disease then a more careful approach should be used in writing these results.

**We agree that it would be ideal to access clinical data to confirm and further classify these self-reported diagnoses. Unfortunately, clinical data are not available. We have added “self-reported” ahead of “kidney disease” in the results and discussion, and we have expanded and reworded the discussion of these results and related limitations (please see above response to Dr. Pendu).**

The associations between FUT2 heterozygosity and gonorrhea, where heterozygotes behaved similarly to non-secretors, is not significant and the relative risk in Figure 1 includes large confidence intervals (from <1 to 2). 

**We have removed the statement regarding gonorrhea.**

Also, the authors should not conclude that there is absence of recessivity since they did not exclude the presence of other null/nonfunctional FUT2 alleles. This hypothesis could be tested by excluding alternative mutations, namely rs1047781 and rs602662.

**Thank you for this suggestion. We have repeated our analysis accounting for these two alternative mutations, and the results are essentially unchanged. This is not surprising because: **

- **The rs1047781A>T mutation is only observed in Asian populations. As expected, it was not observed in our Caucasian population.**
- **The rs602662G>A mutation is highly correlated with the main rs601338 mutation that we have already analyzed. Accounting for this mutation identified an additional 155 non-secretors; the secretor status of the remaining 7427 individuals (98%) did not change.**

 

**We have now mentioned in the Methods section: *“Two other commonly reported non-secretor alleles were considered. The missense variant at rs1047781, described in Asian populations (Ferrer et al. 2009)) was not detected in our Caucasian population. The non-synonymous S258G variant at rs602662 (Barret et al. 2008) was highly correlated with rs601338; incorporating this SNP to define secretor status had no impact on 98% of participants’ phenotype classification, and did not materially change our results.”***

In the analysis of population stratification the authors should explain why they used HapMap populations instead of 1000 Genomes, which includes more samples of the different human groups (European, Africans and Asians) and also have a deeper variation coverage.

**HapMap was used because the analysis of population stratification was completed for the ALSPAC cohort before 1000 Genomes data were available. HapMap is adequate for checking overt ancestry as part of genotyping cleaning, especially because the ALSPAC cohort is 98% Caucasian, and genetic analyses were restricted to Caucasians (this information has now been added to the Methods). We have now cited the 2004 paper that describes this analysis (Pembrey et al.** **https://www.ncbi.nlm.nih.gov/pubmed/15554897** **). Note that for SNP imputation, which was completed more recently, we used the Haplotype Reference Consortium, which is even more fine-scale than 1000 Genomes.**

# Peer review: Referee response for version 1

**DOI:** 10.21956/wellcomeopenres.15935.r33236

**Published:** 2018-06-22

**Authors:** Jacques Le Pendu

**Affiliations:** 1 CRCINA (Center for Research in Cancerology and Immunology Nantes-Angers), Inserm (French National Institute of Health and Medical Research), University of Angers, University of Nantes, Nantes, France

The manuscript by Azad *et al. *describes the search for associations between the FUT2 genotype (or secretor phenotype) with several infectious diseases or chronic conditions from the ALSPAC cohort. Analysis of the rs601338 SNP from over 7500 women of the cohort revealed several significant associations, the two most striking ones linking the nonsecretor phenotype (AA genotype) to mumps and kidney disease. The relationship between the FUT2 AA genotype and mumps may be explained by the use of alpha2,3sialylated motifs as receptors for the MuV that would be hidden by the prior transfer of a fucose, as hypothesized earlier by Tian *et al* ^1^. This constitutes an important independent replication study for that particular association.

 Specific comments:

 - The Tian *et al* study also described an association between mumps and the ABO genotype. Since the A and B blood group enzymes act in concert with the FUT2 enzyme, it would be interesting to analyse the ABO polymorphism in combination with the FUT2 genotype in order to get a better understanding of the genetics of susceptibility to mumps. This would be a significant improvement to the manuscript.

 - A weakness of the study lies in the loosely defined clinical conditions that may limit the ability to uncover significant associations with the FUT2 status. Thus, a borderline association only was observed with non-defined urinary infection although a strong association between the nonsecretor phenotype and recurrent urinary tract infection caused by E. coli uropathogenic strains has been described several times. Likewise, the very interesting association observed between the FUT2 AA genotype and kidney disease leaves the reader unsatisfied since one would like to know the nature of the associated kidney diseases. Considering the above-mentioned infections by E. coli uropathogenic strains, an association with pyelonephritis would be expected. This limitations to the study should be discussed.

 - From a methodological point of view, although I am not a statistician, I wander if a correction for multiple testing would not be necessary since the authors tested for associations between the FUT2 status and 19 independent infectious or chronic conditions. This should be checked and the corrected analysis performed if required.

I have read this submission. I believe that I have an appropriate level of expertise to confirm that it is of an acceptable scientific standard, however I have significant reservations, as outlined above.

## References

[1] TianCHromatkaBKieferAErikssonNNobleSTungJHindsD: Genome-wide association and HLA region fine-mapping studies identify susceptibility loci for multiple common infections. *Nature Communications*.2017;8(1) : 10.1038/s41467-017-00257-5 10.1038/s41467-017-00257-5 PMC560571128928442

# Response

**Published:** 2018-08-14

**Authors:** Meghan Azad

**Affiliations:** University of Manitoba, Canada

**Thank you for your comments, Dr. Le Pendu. Please see our pont-by-point responses below in bold.**

 The manuscript by Azad *et al. *describes the search for associations between the FUT2 genotype (or secretor phenotype) with several infectious diseases or chronic conditions from the ALSPAC cohort. Analysis of the rs601338 SNP from over 7500 women of the cohort revealed several significant associations, the two most striking ones linking the nonsecretor phenotype (AA genotype) to mumps and kidney disease. The relationship between the FUT2 AA genotype and mumps may be explained by the use of alpha2,3sialylated motifs as receptors for the MuV that would be hidden by the prior transfer of a fucose, as hypothesized earlier by Tian *et al* ^1^. This constitutes an important independent replication study for that particular association.

**Thank you for these positive remarks.**

 Specific comments:

 - The Tian *et al* study also described an association between mumps and the ABO genotype. Since the A and B blood group enzymes act in concert with the FUT2 enzyme, it would be interesting to analyse the ABO polymorphism in combination with the FUT2 genotype in order to get a better understanding of the genetics of susceptibility to mumps. This would be a significant improvement to the manuscript.

**Thank you for this suggestion. We have now incorporated clinical blood group data in our analysis. This is described in the Methods, Results (new Table 3) and Discussion:**

***(METHODS) “ABO blood group was collected from clinical records for the majority of participants (N=6,757).” and “Multivariable models were also used to mutually adjust for FUT2 secretor status and ABO blood group and to formally test for interaction between these two factors.”***

***(RESULTS) “Since secretor status determines the ability to secrete blood group antigens, we also explored the impact of ABO blood group on associations observed for mumps infection and kidney disease ( SEE NEW TABLE 3: Mutually-adjusted associations of FUT2 secretor status and ABO blood group with mumps infection and kidney disease in the ALSPAC cohort). For both conditions, the effect estimate for FUT2 secretor status was essentially unchanged following adjustment for ABO blood group and there was no evidence of interaction between FUT2 and ABO blood group (p for interaction: 0.60 for mumps, 0.57 for kidney disease). Independent of FUT2 secretor status, women with type AB blood had an increased risk of mumps infection (59.4%) compared to women with type A, B, or O blood (52.2%, 52.0%, 52.9%, respectively; adjusted RR 1.15, 95% CI: 1.03, 1.28 for AB vs O, p=0.01). ABO blood group was not associated with kidney disease.”***

***(DISCUSSION) “Tian et al. also reported an association between the ABO gene and mumps infection, and suggested that ABO antigens may disrupt binding of the mumps virus to host cell receptors. Our results using clinical ABO blood group data support and extend this finding by confirming an association and specifically identifying blood type AB as a risk factor for mumps infection. Since FUT2 secretor status determines whether ABO antigens are secreted into body fluids and onto cell surfaces, we hypothesized that secretor status and blood type may interact to influence susceptibility to mumps infection; however, we found no evidence of this interaction. Thus, our study suggests that FUT2 genotype and ABO blood group are independently associated with mumps infection, with increased risk among non-secretors and blood type AB.” … “Power was limited for interaction analyses.”***

 - A weakness of the study lies in the loosely defined clinical conditions that may limit the ability to uncover significant associations with the FUT2 status. Thus, a borderline association only was observed with non-defined urinary infection although a strong association between the nonsecretor phenotype and recurrent urinary tract infection caused by E. coli uropathogenic strains has been described several times. Likewise, the very interesting association observed between the FUT2 AA genotype and kidney disease leaves the reader unsatisfied since one would like to know the nature of the associated kidney diseases. Considering the above-mentioned infections by E. coli uropathogenic strains, an association with pyelonephritis would be expected. This limitations to the study should be discussed.

**We agree that the lack of precise clinical information is a limitation of our study.  We have expanded our discussion of this limitation by adding the following sentence:**

***“We could not identify the specific pathogens responsible for urinary infections, and we lacked clinical data to confirm, classify and define the etiology of multifactorial disorders (such as allergies and kidney disease).”***

**We have also expanded our discussion of the results for kidney disease and urinary infections, citing evidence for uropathogenic E. coli and acute pyelonephritis.  Thank you for this helpful suggestion. The revised paragraph now reads:**

***“Our study also provides new evidence that non-secretors may be predisposed to kidney disease (RR, 1.39; 95% CI, 1.11–1.75), although we lacked clinical information to confirm and classify this self-reported diagnosis. To our knowledge, the FUT2 genotype has not previously been associated with kidney disease in the general population, although some studies have used traditional blood group assays to evaluate secretor status in patients with pyelonephritis (kidney inflammation, typically due to bacterial infection).  One study of women with acute uncomplicated pyelonephritis found that non-secretor status was significantly higher in these patients than in the general population (Ishitoya et al. 2002), and another found that renal scarring in girls with recurrent pyelonephritis was more common in non-secretors than secretors ^27^. It has been hypothesized that these associations reflect an increased susceptibility to uropathogenic Escherichia coli infection among non-secretors, resulting from the enhanced expression of preferred binding receptors in the vaginal epithelium and kidneys of non-secretor women (Stapleton et al. 1992; Stapleton et al. 1998). Notably, in our study, the association between FUT2 genotype and self-reported kidney disease appeared to be independent of self-reported urinary infections. However, there are multiple clinically-distinct causes of “kidney disease” and “urinary infections”, and we lacked clinical information to define the etiology of these conditions in our study. Thus, additional research is needed to replicate our observations with confirmed and clinically-defined kidney disease and urinary infections, and to examine the possible relationship between these conditions and FUT2 genotype.”***

 - From a methodological point of view, although I am not a statistician, I wander if a correction for multiple testing would not be necessary since the authors tested for associations between the FUT2 status and 19 independent infectious or chronic conditions. This should be checked and the corrected analysis performed if required.

**Thank you for this suggestion. We considered correcting for multiple testing, but ultimately decided against this because the conditions may not be independent of each other (especially if similar or related FUT2-depndent mechanisms are influencing multiple conditions).**
